# Deontic-doxastic belief revision and linear system model

**DOI:** 10.3389/fpsyg.2022.948330

**Published:** 2022-08-11

**Authors:** Andrea Vestrucci

**Affiliations:** ^1^Faculty of Information Systems and Applied Computer Sciences, University of Bamberg, Bavaria, Germany; ^2^Starr King School, Oakland, CA, United States

**Keywords:** belief revision, Dynamic Epistemic Logic, AGM theory, doxastic voluntarism, deontic logic, doxastic logic, belief models, logic of belief revision

## Introduction

The article presents a doxastic-nested-deontic formalization of epistemic deontology (Feldman, 2000; Forrai, 2021) for static and dynamic belief revision, in AGM theory (and extensions) and Dynamic Epistemic Logic, respectively. The article also introduces a linear system model for beliefs^1^.

Doxastic and deontic logics axiomatize propositions about beliefs (“it is believed that”) and prescriptions (“it is obligatory that”), respectively. They belong to the family of modal logics.

Static and dynamic belief revisions follow from adding conflicting information to a belief database: in the static setting the doxastic value of the information is fixed (revision is non-iterated); in the dynamic setting information can be revised (revision can be iterated). In light of this, static belief revision might seem incompatible with belief update (Katsuno and Mendelzon, 1992) since update deals with information change (Seitz et al., 2018). This position has been variously challenged (Friedman and Halpern, 1994; Peppas and Williams, 1995; Gabbay, 1999; Aucher, 2004). Belief revision theories do relate to models for database update (Val and Shoham, 1994; Williams, 1997; Ditmarsch et al., 2008).

The article's outputs address: doxastic voluntarism; a paradox in strong epistemic deontology; the specificity of religious beliefs (Oviedo and Szocik, 2020).

## Static belief revision

Beliefs are elements of a set B (Alchourrón et al., 1985; Gärdenfors, 1988) over which three relations are defined: (1) logical consequence “⊢” (Gärdenfors, 1984; Alchourrón et al., 1985); (2) epistemic entrenchment “≼” (Gärdenfors and Makinson, 1988), and (3) spheres inclusion “≥” (Grove, 1988).

Concerning 1, elements of B are logical consequences of other elements (e.g., believing that tomorrow will rain follows from believing in the reliability of weather forecasts).


$$\begin{array}{l} B=Cn\left(B\right)=\left\{\alpha :B\;⊢\;\alpha \right\} \end{array}$$

(Huber, 2013; Hansson, 2022).

In case of a new information ϑ contradicting some elements of B, B is revised (“∗”) by clearing B from all elements contradicted by ϑ, and adding ϑ:


$$\begin{array}{l} B*\vartheta =Cn\left(B-\neg \vartheta \cup \{\vartheta \}\right) \end{array}$$

(Levi, 1977).

Concerning 2, entrenchment is a preorder on B (Peppas, 2008) based on belief firmness: the more a belief is entrenched, the more it costs to give it up. This applies also to logical consequences; thus 1 and 2 are related: α ⊢ β → α ≼ β (Dominance postulate). Belief revision deals with clearing B from anything that is less or equally entrenched than all elements contradicted by ϑ, and adding ϑ.


$$\begin{array}{l} B*\vartheta =Cn\left(\left\{\psi \in \;B:\neg \vartheta ≺\psi \right\}\cup \left\{\vartheta \right\}\right). \end{array}$$

Revision consists in the “minimal mutilation” (Rott, 2000; Leitgeb, 2010) of B [keeping as much old beliefs as possible (Ditmarsch et al., 2008)], and the addition of ϑ.

Concerning 3, worlds *w* in which elements from B are true are placed on spheres ordered per inclusion. Given a Kripke model *M*, ${\left[B\right]}_{M}=\left\{w\in W^{M}:\;M,w⊨\varphi \;\forall \varphi \in B\right\}$. Inclusion can be grasped as *plausibility* order (Peppas, 2008): the most plausible possible worlds are located on spheres with the least radius. Thus, 3 is related to 2: φ ≼ ψ ≅ [φ]_*M*_ ≥ [ψ]_*M*_. Considering ${\left[\vartheta \right]}_{M}=\left\{w\in W^{M}:\;M,w⊨\vartheta \right\}$, agent *a*'s belief in φ conditioned on ϑ (“$B_{a}^{\vartheta }\varphi$”) is true in the minimal-radius spheres (i.e., most plausible worlds) in which ϑ is also true. By simplifying Baltag and Renne, 2016:


$$\begin{array}{l} M,w⊨B_{a}^{\vartheta }\varphi \in B*\vartheta \equiv min_{a}\left({\left[\vartheta \right]}_{M}\right)\subseteq {\left[\varphi \right]}_{M}. \end{array}$$

This corresponds to making ϕ a *safe* belief (Baltag et al., 2008):


$$\begin{array}{l} M,w⊨B_{a}^{\vartheta }\varphi \in B*\vartheta \equiv M,w⊨{□}_{a}\varphi .\; \end{array}$$

Thus, the formula for epistemic deontology of belief revision in a static setting corresponds to:


$$\begin{array}{l} \left(\left({□}_{a}\vartheta \wedge \exists x\in B_{a}:\vartheta ⊬x\right)\to \right)\;◇\text{O}(\forall \phi \in B_{a},\;\phi \to {□}_{a}\varphi ).\; \end{array}$$

Since $B_{a}^{\vartheta }\varphi$ is a doxastic conditional, ◇**O** is a conditioned obligation presupposing that agent *a* has at least a safe belief on ϑ, and that ϑ is in a negative relation with at least one element of B. The formula represents the duty to increase the epistemic degree of set B: it formalizes Kant's “*Sapere aude!*” (Kant, 2013).

The nested formula applies to negative doxastic voluntarism (NDV), the idea that we have control not over belief formation but over belief withdrawal (Rott, 2017). The formula translates “belief withdrawal” into “epistemic-degree-increase duty,” and it associates the notion of “negative control” to the whole spectrum of duty realizations, including duty *non*-realization; thus, voluntarism pertains also to the refusal of epistemic degree increase. Moreover, since the duty is conditional, the formula expands NDV to include (or even presuppose) belief expansion (□*_a_*ϑ).

## Dynamic belief revision

In static belief revision, information ϑ is included in the revised set (Success postulate: ϑ∈B∗ϑ). Thus, static revision assumes the epistemic value of ϑ to be unchangeable. This is problematic, e.g., in the case of Moore sentences involving higher-order beliefs (Baltag et al., 2008). To amend this, ϑ shall be considered susceptible of revision too. Research in dynamic belief revision distinguishes at least three epistemic degrees of ϑ (van Benthem, 2007; Baltag and Smets, 2009; Baltag et al., 2014): (1) ϑ is “hard information” issued from an infallible source: it is neither revisable nor revocable; (2) ϑ is “soft information” from a fallible, yet highly reliable source; (3) ϑ is “soft information” from a barely trusted source (truthfulness can be easily given up).

To these three doxastic degrees correspond three types of dynamic belief revision:

1. Radical revision [!ϑ]: it eliminates all ¬ϑ-worlds and the previous plausibility order is preserved between the remaining worlds.
2. Lexicographic (radical) revision [⇑ϑ]: all ϑ-worlds are made more plausible than ¬ϑ-words, and the rest of the order is unchanged.
3. Conservative (neutral) revision [↑ϑ]: the most plausible ϑ-words become the most plausible worlds overall, and all rest is unchanged.

Thus, the formula for the epistemic deontology of belief revision in a dynamic setting corresponds to (the lexicographic formula; van Benthem, 2011 is a generalization of the conservative one):


$$\begin{array}{l} □/◇\text{O}\left(\begin{array}{c} \left[!\vartheta \right]B_{a}\varphi \equiv \left(\vartheta \to B_{a}^{\vartheta }\left(\left[!\vartheta \right]\varphi \right)\right)\\ M,w⊨\left[⇑\vartheta \right]B_{a}\varphi \equiv M⇑\vartheta ,w⊨B_{a}\varphi \\ \left[↑\vartheta \right]B_{a}\varphi \end{array}\right) \end{array}$$

The deontic operator might be not conditioned since the doxastic conditions for revision are *within* the obligation. This would introduce to a strong epistemic deontology: under no condition a belief is allowed to be held if no sufficient evidence supports it.

This leads to a paradox in strong epistemic deontology. Let's assume two scenarios: 1. the revision process halts, 2. it does not halt. In 1, the revision halts because a belief has received sufficient evidence to be no longer revisable. Thus, it is not even a (safe) belief: it is infallible and indefeasible *knowledge* resisting any information (true or false) (Baltag and Smets, 2008). In 2, the reiterated halt delay means that the collection of evidence never ends: the belief is never allowed to be held. Thus, from a strong epistemic deontology, no belief is ever legitimate, regardless of the doxastic degree of it: either a belief is transformed into knowledge, or it is never sufficiently justified. Hence, the paradox: believing is always wrong *for the fact itself of believing*.

The deontic encapsulation of dynamic belief revision might address this paradox by including not only belief revision, but also information ϑ in the *deontic* environment. The duty of belief revision is not unconditioned, but conditioned by the duty of evaluating the object itself of duty (collecting ϑ) either positively or negatively. This might include the rejection or neglection of information ϑ as forms of epistemic deontology satisfaction.

## Linear system model

The aforementioned theories conceive beliefs as elements of a set. This set model imposes at least three requirements: (1) The elements of a belief set must be orderable according to some (pre)orders; (2) The belief set must be somehow coherent, and belief revision corresponds to the maximal preservation of this coherency; (3) A new information is needed, which contradicts at least one belief. The weight of the revision work is proportional to the number of beliefs connected to the new information, and to the negativity of this connection.

Do these three requirements apply to all belief set revisions? If we take the case of religious beliefs, then: (1) An ordering relation implies a comparability between the set elements which at its turn implies a homogeneity of the elements' epistemic bases. However, religious beliefs cover different epistemic spheres: metaphysical, moral, aesthetic, pragmatic, etc. It's not clear how beliefs referring to such different epistemic spheres can be fully comparable. (2) The issue of theodicy is evidence against the (at least *prima facie*) coherency of religious beliefs since theodicy tries to address the incompatibility between the belief in divine omnipotence and the belief in divine justice. (3) The revision of a religious set may start not only from external information, but also from introspection, i.e., the internal evaluation and investigation of one's faith.

Thus, I propose an alternative model, in which beliefs are elements of a system of linear equations. This linear system model has at least two advantages compared to the set model:

1. Bottom-up organization. In the set model, a belief's relevance depends on its being an element of a set, i.e., the belief characteristics are deduced from the set definition. This is why in the set model beliefs constitute a coherent unity and are ordainable: conditions 1 and 2 follow from the application of the set model to beliefs (and their revision). In the linear system model, the solution of the system is given by the linear equations (the elements) constituting the system. Thus, the belief's characteristics precede (and not follow from) the system including them: rather than selecting beliefs in light of a certain model (a certain definition of belief set), the model is constructed and constantly readjusted in light of the elements we aim to investigate. This bottom-up organization respects the epistemic “matter” by building the model *upon* this matter.
2. Representation of belief *stratification*. Beliefs are stratified vertically and horizontally. The vertical stratification is the succession of beliefs, represented by the order of the equations in the linear system; this succession is not necessarily a preorder since the equations' order does not change the system's solution. However, the vertical stratification has a *procedural* function: it eases the substitution of the variables that are gradually known. Moreover, the system might allocate different epistemic spheres in different vertical strata, thus not overlapping epistemically distinct beliefs. The horizontal stratification is the composition of a belief as a sum of *sub-beliefs*: each sub-belief is a part of the greater belief, and their order in the summation corresponds to their relevance within the whole belief. For example, the belief in the 10 commandments is composed by the sub-beliefs in all single commandments, each sub-belief doxastically introducing the successive.

This linear system model is:


$$\begin{array}{l} R=\{\begin{array}{c} a_{1,1}{□}_{1}+a_{1,2}{□}_{2}+a_{1,3}{□}_{3}+\ldots +a_{1,n}{□}_{n}=w_{1}\\ \ldots \\ a_{m,1}{□}_{1}+a_{m,2}{□}_{2}+a_{m,3}{□}_{3}+\ldots +a_{m,n}{□}_{n}=w_{m} \end{array} \end{array}$$

$$\begin{array}{l} R=\overset{m}{\underset{i=1}{⋂}}\overset{n}{\underset{j=1}{\sum }}a_{i,j}{□}_{j}=\overset{m}{\underset{i=1}{⋂}}w_{i}\; \end{array}$$

A system *R* represents the vertical stratification of *m* beliefs: it is the intersection of *m* polynomial equations with *n* variables. In each equation, the coefficient *a*_*i, j*_ is the content of a belief or sub-belief, e.g., an equation with ten coefficients might represent the belief in the ten commandments. The variable □_*j*_ is the doxastic value associated to the belief content in position *j*. The doxastic value is the same for all coefficients in position *j* since it follows the horizontal stratification: a sub-belief in *j* is the doxastic “step” to reach the sub-beliefs in positions *k* > *j*. The constant term *w*_*i*_ expresses the possible world plausibility of the entire polynomial.

This model permits a more “economic” belief revision. In the set model (for both static and dynamic scenarios), revision consists in a modification of the set structure: a subset is eliminated or displaced in light of new information. In the linear system model, the elimination of a belief (equation) does not necessarily affect the system: the condition is for the number of equations to be at least equal to the number of doxastic values; one can obtain this by readjusting some coefficients (sub-beliefs), e.g., expunging the *filioque* belief without touching other religious-metaphysical beliefs, but maybe modifying some religious-aesthetic beliefs.

This model also permits a simpler procedure to compare different belief systems. For example, an orthodox and a non-orthodox Christian might have belief systems (resp. *R*_1_ and *R*_2_) which differ for the third equation (non-*filioque* in *R*_1_, in bold), but are identical for the rest.

$$\begin{array}{l} R_{1}=\{\begin{array}{c} a_{1,1}{□}_{1}+a_{1,2}{□}_{2}+a_{1,3}{□}_{3}+\ldots +a_{1,10}{□}_{10}=w_{1}\\ b_{2,1}{□}_{1}=w_{2}\\ c_{3,1}{□}_{1}+{0{□}_{2}+c}_{3,3}{□}_{3}\;=\;w_{3}\\ \ldots \end{array} \end{array}$$

$$\begin{array}{l} R_{2}=\{\begin{array}{c} a_{1,1}{□}_{1}+a_{1,2}{□}_{2}+a_{1,3}{□}_{3}+\ldots +a_{1,10}{□}_{10}=w_{1}\\ b_{2,1}{□}_{1}=w_{2}\\ c_{3,1}{□}_{1}+c_{3,2}{□}_{2}+c_{3,3}{□}_{3}\;=\;w_{3}\\ \ldots \end{array} \end{array}$$

Matrix form is even clearer:

$$\begin{array}{l} R_{1}=\left[\begin{array}{ccccc} a_{1,1} & a_{1,2} & a_{1,3} & \cdots & a_{1,10}\\ b_{2,1} & 0 & 0 & \cdots & 0\\ c_{3,1} & 0 & c_{3,3} & \cdots & 0\\ \vdots & \vdots & \vdots & \vdots & \ddots \end{array}\right]\\ R_{2}=\left[\begin{array}{ccccc} a_{1,1} & a_{1,2} & a_{1,3} & \cdots & a_{1,10}\\ b_{2,1} & 0 & 0 & \cdots & 0\\ c_{3,1} & c_{3,2} & c_{3,3} & \cdots & 0\\ \vdots & \vdots & \vdots & \vdots & \ddots \end{array}\right] \end{array}$$

The linear system model presents an intuitive approach to synthetically grasp a relationship between belief systems. Thus, the model might better capture the limits and extent of ecumenical and interreligious dialogues.

## Discussion

Aspects of future investigation include: establishing the doxastic-nested-deontic grammar; assessing the approach it provides to doxastic voluntarism; presenting a deontic investigation of the epistemic deontology paradox; deepening the potentialities and weaknesses of the linear system model for beliefs; exploring belief translatability from the linear model to the set model and vice-versa.

## Author contributions

The author confirms being the sole contributor of this work and has approved it for publication.

## Funding

This paper was funded by Dr. Rüdiger Seitz, via the Volkswagen Foundation, Siemens Healthineers, and the Betz Foundation.

## Conflict of interest

The author declares that the research was conducted in the absence of any commercial or financial relationships that could be construed as a potential conflict of interest.

## Publisher's note

All claims expressed in this article are solely those of the authors and do not necessarily represent those of their affiliated organizations, or those of the publisher, the editors and the reviewers. Any product that may be evaluated in this article, or claim that may be made by its manufacturer, is not guaranteed or endorsed by the publisher.

## Footnotes

^1^The limits of this article do not allow consideration of other belief revision theories – e.g., ranking theory (Spohn, 1988, 2012; Huber, 2006, 2021) and Bayesian model (Brown et al., 2019) – nor discussion of AGM theory being an idealization of actual human doxastic agents in light of the logical, epistemological, and empirical simplifications involved in AGM (Wassermann, 1999; Berto, 2019). However, this idealization is useful to formalize belief revision (Hansson, 2022), thus paving the way to models more adherent to real doxastic situations, such as Dynamic Epistemic Logic (Section 3) and the linear system model (Section 4). Any adherence is nevertheless affected by the distinction between model and modeled object. AGM's clarity and logical and computational versatility (Delgrande et al., 2013; Spurkeland et al., 2013) make it a good candidate to introduce the doxastic-nested-deontic grammar in this article.
